# The relationship between fundamental motor skills and physical fitness in preschoolers: a short-term longitudinal study

**DOI:** 10.3389/fpsyg.2023.1270888

**Published:** 2023-09-14

**Authors:** Jiebo Chen, Wenjing Song, Xing Zhao, Hu Lou, Dongmei Luo

**Affiliations:** ^1^Department of Sports Science, Nantong University, Nantong, China; ^2^Department of Kinesiology, Beijing Sport University, Beijing, China

**Keywords:** fundamental motor skills, physical fitness, preschoolers, longitudinal data, cross-lagged model

## Abstract

**Purpose:**

Physical fitness and fundamental motor skills are two important aspects for the healthy development of preschoolers. Despite the growing interest in clarifying their relationship, the scarcity of longitudinal studies prevents us from understanding causality.

**Method:**

This study employed a cross-lagged model with two time points to investigate the bidirectional relationship between these two aspects. A total of 174 preschoolers (54.0% girls) from 3 to 6 years old (*M* = 3.96 ± 0.47) were surveyed, they were recruited by convenience from two kindergartens in Beijing, China, and their physical fitness (via CNPFDSM-EC) and fundamental motor skills (via TGMD-3) were tracked over a period of 6 months.

**Results:**

The findings revealed a bidirectional predictive effect. The predictive strength of flexibility was found to be lower than other physical fitness aspects, while locomotor skills demonstrated a higher predictive strength than object control skills.

**Conclusion:**

This study indicates that physical fitness and fundamental motor skills mutually enhance each other in young children, and both should be emphasized in preschool sports education.

## 1. Introduction

Fundamental motor skills and physical fitness are crucial factors in fostering positive trajectories of health over time. Fundamental motor skills refer to basic, non-naturally occurring, and learnable movement patterns (Haywood and Getchell, 2014). In early life, mastering a broad range of these skills is beneficial for future learning of more complex motor skills, active and continuous participation in various sports, and the development of a lifelong exercise habit (Henrique et al., 2016). Furthermore, during this developmental period, fundamental motor skills are positively correlated with children’s physical activity, healthy weight status, and even their academic performance (Robinson et al., 2015; Engel et al., 2018; de Waal, 2019). Physical fitness refers to an individual’s capacity to undertake physical activity. Its components include cardiorespiratory fitness, muscular strength, speed-agility and flexibility (Caspersen et al., 1985), and it is a health and well-being indicator even from a young age (Ortega et al., 2008). Moreover, physical fitness is thought to have a positive effect on a range of psychological indicators, such as cognition, depression, and self-esteem (Cadenas-Sanchez et al., 2021).

Fundamental motor skills and physical fitness are correlated in early childhood (Utesch et al., 2019). The conceptual framework presented by Stodden (2008) explains that the acquisition of motor skill competence in fundamental motor skills in early childhood (2–5 years of age) serves to enhance physical fitness. As the child ages, this relationship evolves into a bidirectional one in mid to late childhood. Similarly, Hulteen et al. (2018) propose a model that delineates the progression of motor skills over time. This model illustrates that skill development can either be hindered or enhanced by physical and psychological factors such as physical fitness and perceived competence. Both models emphasize the importance of understanding the development of fundamental motor skills and physical fitness, as well as their influence on each other.

Although both models hypothesize a relationship between fundamental motor skills and physical fitness during early childhood, the current research evidence is quite limited. This is supported by the results of a meta-analysis, which underscores the lack of studies investigating the relationship between fundamental motor skills and physical fitness in children younger than 7–8 years of age (Utesch et al., 2019). Only two cross-sectional studies have explored the correlation between these two aspects among preschoolers. Frith and Loprinzi (2019) demonstrated a positive correlation between motor skills and musculoskeletal endurance (measured via the plank test), and Wang et al. (2019) supported a low to moderate positive association between fundamental motor skills and physical fitness. However, due to the cross-sectional nature of these studies, it’s impossible to determine causality in their co-development. Longitudinal studies provide the potential to uncover their dynamic and reciprocal relationship. This capability will assist researchers and practitioners in understanding how to stimulate and nurture physical fitness and motor competence more effectively. The ultimate goal is to improve health outcomes and promote lifelong physical activity.

Thus, the goal of this study is to employ a longitudinal study design, comprising two time points, to explore the relationship between physical fitness and fundamental motor skills through a cross-lagged model, after controlling for confounding factors such as age, gender and BMI. Our hypothesis is that the relationship between these two aspects at the preschool age is bidirectional and mutually reinforcing.

## 2. Materials and methods

### 2.1. Participants

An observational longitudinal design was used to study 174 typically developed children (54.0% girls) from 3 to 6 years old (*M* = 3.96 ± 0.47) at baseline. Participants were recruited by convenience from two kindergartens in Beijing, China. Data were collected two time points, the baseline test (T1) was conducted from November to December 2020, and the follow-up test (T2) was conducted at a 6-month interval, from May to June 2021. A total of 212 children participated in T1, and 174 participated in T2 (the lower number being a result of absences, illnesses and transfers), with an attrition rate of 17.5%. Analysis of the attrition sample, chi-square and t-test results suggested no significant difference between children who continued to participate in the study and those who did not, in terms of gender (χ^2^ = 2.71, *p* = 0.23); age (t = 0.97, *p* = 0.45) and aspects of physical fitness and fundamental motor skills (*p* > 0.05). This indicated random attrition. Parents of all participants signed the Parental Informed Consent Form to indicate their consent to participate in the study.

### 2.2. Instruments

*Fundamental motor skills* were assessed using the third edition of the Test of Gross Motor Development (TGMD-3), a process-oriented test that contains locomotor and object control subtests (Ulrich, 2013) The former subtest comprised 6 skills: running, horizontal jump, hop, gallop, slide, and skip; and the latter 7: two-handed strike of a stationary ball, forehand strike of self-bounced ball, one hand stationary dribble, overhand throw, underhand throw, two-handed catch, and kicking a stationary ball. Each skill in TGMD-3 was tested twice and scored according to 3 to 5 criteria, with 1 point for meeting one criterion and 0 points for meeting none. The original score was 46 for locomotor skills, 54 for object control motor skills, and 100 for total score. This scale has acceptable internal consistency and test–retest reliability for Chinese children (Ning et al., 2016a; Diao et al., 2018).

*Physical fitness* was assessed using the early childhood section of the China National Physical Fitness Determination Standard Manual(CNPFDSM-EC), formulated by the State General Administration of Sports (Zhang et al., 2017). The test comprised 6 items: 10-meter shuttle run, horizontal jump, tennis ball throwing, continuous jumping with both feet, sit-and-reach, and balance beam walking. All items were measured twice, and the best score taken. In addition to physical fitness, the height and weight of the preschoolers were measured, and the Body Mass Index (BMI = weight/[height^2^]) was calculated. The classification of overweight and obese used in this study is based on the BMI criteria established by the International Obesity Working Group (Cole, 2000).

### 2.3. Procedure

All tests were conducted in the playgrounds of the respective schools and lasted approximately 30–45 min per child.

Tests of fundamental motor skills were performed by two trained research assistants, one of whom was responsible for demonstrating and testing, and the other for recording the preschoolers’ performances using a video camera. The testing protocol began with a single demonstration by the research assistant, followed by one practice trial. If the child’s performance during the trial was deemed incorrect, an additional demonstration was provided. Subsequently, two formal tests were administered. After the test, a trained observer assessed the child’s performance via video. The observer had a previously established 93% interrater agreement with a trained researcher.

Physical fitness, height and weight tests were administered by three additional trained research assistants. Each assistant had a distinct role: one conducted the tests, another ensured the children’s safety, and the third recorded the test results. To ensure both testing efficiency and adherence to the principles of static and dynamic performance during the testing process, a sequence was established: balance beam walking, 10-meter shuttle run, standing long jump, tennis ball throwing, continuous jumping with both feet, sit-and-reach. The final measurements were height and weight.

### 2.4. Statistical analyses

First, descriptive statistics were obtained for each variable, and the age and gender characteristics of BMI, physical fitness and fundamental motor skills were presented by a two-way analysis of variance (two-way ANOVA), with the changes in the development of each variable over time presented in a paired t-test. Second, to examine the correlation between BMI, physical fitness and fundamental motor skills at two time points, correlation indexes were calculated through Pearson’s correlation coefficient. Finally, cross-lagged models were used, controlling for variables such as age, gender and BMI, to further confirm the chronological order and causality between physical fitness and fundamental motor skills.

The two-way ANOVA, paired t-test and correlation analysis were performed in SPSS 18.0, and the cross-lagged model test in Mplus 8.3. The fit index and acceptable critical values used for the cross-lagged model assessment were χ2, df (χ2 / df < 2), CFI (> 0.90), TLI (> 0.90), RMSEA (< 0.08), and SRMR (< 0.08), respectively, as used in previous studies (Hu and Bentler, 1999).

## 3. Results

### 3.1. Descriptive data and correlations

Before examining the cross-lagged model, we tested the descriptive data and correlation matrix. The results of the two-way ANOVA showed that the main effects of age and gender were significant in both T1 and T2, but there was no interaction between the variables (see Table 1). For this reason, only the separate effects of age and gender will be analyzed.

**Table 1:** Main effects and interaction analysis of age and gender.

Time point	Age		Gender		Age*Gender	
	*F*	*p*	*F*	*p*	*F*	*p*
T1	7.762	< 0.001	3.297	0.001	1.629	0.113
T2	4.276	< 0.001	4.226	< 0.001	0.985	0.470

T1 is the time point of the baseline test; T2 is the time point of the follow-up test, 6 months later.

At T1, the main effects of age on BMI and sit-and-reach were not significant, while the remaining indicators of physical fitness and fundamental motor skills had a significant main effect and increased with age. The main effect of gender was significant only in tennis ball throwing and object control skills, where boys scored significantly higher (*F* = 23.121, *p* < 0.001, η_p_^2^ = 0.171; and *F* = 11.181, *p* < 0.001, η_p_^2^ = 0.134 respectively) (see Table 2). At T2, the main effects of age on BMI and object control skills were not significant, the remaining indicators also increased with age, with a significant main effect. The main effect of gender was significant only in tennis ball throwing, object control skills and sit-and-reach. In the former two, boys scored significantly higher (*F* = 16.690, *p* < 0.001, η_p_^2^ = 0.152; and *F* = 16.064, *p* < 0.001, η_p_^2^ = 0.147 respectively), but had lower scores in sit-and-reach (*F* = 11.181, *p* = 0.001, η_p_^2^ = 0.134) (see Table 3). The 6-month follow-up data suggested that the BMI of participants did not change over time (*t* = 1.109, *p* = 0.270), while the other indicators improved significantly (see Table 4).

**Table 2:** Descriptive statistics at T1.

Indicator	Age 3.5		Age 4.5		*p*	
	Boys (*n* = 40)	Girls (*n* = 48)	Boys (*n* = 40)	Girls (*n* = 46)	Age	Gender
BMI (kg/m^2^)	16.4 ± 1.26	15.7 ± 1.17	15.8 ± 1.22	15.8 ± 1.80	0.540	0.306
Overweight (*n*, %)	2, 1.15%	3, 1.72%	0	1, 0.57%	–	–
Obese (*n*, %)	2, 1.15%	1, 0.57%	0	0	–	–
Physical fitness						
Horizontal jump (cm)	68.1 ± 15.33	70.9 ± 14.24	86.3 ± 21.14	83.3 ± 18.84	**< 0.001**	0.983
Tennis ball throwing (m)	3.8 ± 1.52	2.9 ± 0.67	4.9 ± 1.84	3.6 ± 0.87	**0.003**	**< 0.001**
Sit-and-reach (cm)	8.9 ± 2.99	11.9 ± 3.74	10.2 ± 4.11	10.2 ± 4.60	0.802	0.112
10-meter shuttle run (s)	9.1 ± 1.26	9.3 ± 1.21	7.5 ± 0.77	7.7 ± 0.72	**< 0.001**	0.272
Balance beam walking (s)	21.2 ± 13.63	14.5 ± 7.13	7.2 ± 4.14	9.4 ± 4.38	**< 0.001**	0.234
Continuous jumping with both feet (s)	7.3 ± 2.70	7.8 ± 3.77	7.1 ± 2.62	5.9 ± 1.21	0.099	0.593
FMS						
Locomotor skills	17.3 ± 8.24	20.6 ± 6.59	27.4 ± 6.29	27.7 ± 5.65	**< 0.001**	0.245
Object control skills	19.6 ± 4.01	15.7 ± 4.59	21.6 ± 5.03	18.9 ± 3.54	**0.012**	**0.001**
Total FMS score	36.9 ± 10.05	36.4 ± 7.85	49.1 ± 10.47	46.6 ± 7.75	**< 0.001**	0.471

The bold values represent that *p* - values are statistically significant.

**Table 3:** Descriptive statistics at T2.

Indicator	Age 4.0		Age 5.0		*p*	
	Boys (*n* = 40)	Girls (*n* = 48)	Boys (*n* = 40)	Girls (*n* = 46)	Age	Gender
BMI (kg/m^2^)	15.7 ± 1.40	15.7 ± 1.11	15.9 ± 1.44	15.5 ± 1.61	0.869	0.464
Overweight (*n*, %)	1, 0.57%	1, 0.57%	1, 0.57%	1, 0.57%	–	–
Obese (*n*, %)	4, 2.30%	1, 0.57%	0	0	–	–
Physical fitness						
Horizontal jump (cm)	93.6 ± 13.14	87.8 ± 15.78	104.3 ± 15.01	105.8 ± 13.20	**< 0.001**	0.470
Tennis ball throwing (m)	5.1 ± 1.30	4.1 ± 0.94	6.3 ± 1.43	5.3 ± 1.21	**< 0.001**	**< 0.001**
sit-and-reach (cm)	13.2 ± 3.78	16.8 ± 2.99	12.0 ± 4.32	14.2 ± 3.12	**0.010**	**< 0.001**
10-meter shuttle run (s)	7.4 ± 0.54	7.5 ± 0.80	6.7 ± 0.62	6.9 ± 0.30	**< 0.001**	0.250
Balance beam walking (s)	8.1 ± 4.43	8.2 ± 3.91	6.7 ± 4.31	6.0 ± 1.79	**0.021**	0.690
Continuous jumping with both feet (s)	6.9 ± 1.26	7.2 ± 1.81	6.2 ± 1.20	6.0 ± 0.63	**< 0.001**	0.837
FMS						
Locomotor skills	24.2 ± 6.48	26.6 ± 6.33	27.9 ± 5.35	28.3 ± 5.19	**0.027**	0.240
Object control skills	20.6 ± 5.64	18.2 ± 7.35	24.4 ± 8.06	19.7 ± 5.51	0.058	**0.010**
Total FMS score	44.8 ± 10.46	44.8 ± 10.59	52.3 ± 11.62	47.7 ± 8.23	**0.014**	0.276

The bold values represent that *p* - values are statistically significant.

**Table 4:** Changes between T1 and T2 data.

	T1	T2	Variation	95%CI	*p*
Body shape					
Height (cm)	105.0 ± 4.54	109.3 ± 4.79	4.2 ± 4.21	(3.4, 5.1)	**<0.001**
Weight (kg)	17.5 ± 2.46	18.8 ± 2.65	1.3 ± 2.25	(0.9, 1.8)	**<0.001**
BMI (kg/m^2^)	15.8 ± 1.41	15.7 ± 1.39	−0.1 ± 1.24	(−0.4, 0.1)	0.270
Physical fitness					
Horizontal jump (cm)	72.1 ± 21.81	98.3 ± 16.11	26.2 ± 20.34	(22.0, 34.0)	**<0.001**
Tennis ball throwing (m)	3.5 ± 1.36	5.2 ± 1.44	1.7 ± 1.72	(1.3, 2.0)	**<0.001**
Sit-and-reach (cm)	10.1 ± 3.92	14.1 ± 3.90	4.0 ± 4.69	(3.0, 4.9)	**<0.001**
10-meter shuttle run (s)	8.7 ± 1.44	7.1 ± 0.66	−1.6 ± 1.31	(−1.8, −1.3)	**<0.001**
Balance beam walking (s)	13.4 ± 9.11	7.3 ± 4.24	−6.1 ± 8.04	(−7.8, −4.4)	**<0.001**
Continuous jumping with both feet (s)	7.7 ± 3.37	6.6 ± 1.80	−1.1 ± 3.26	(−1.8, −0.4)	**0.002**
FMS					
Locomotor skills	22.6 ± 7.81	26.8 ± 5.99	4.1 ± 8.63	(2.4, 5.9)	**<0.001**
Object control skills	17.8 ± 5.24	20.6 ± 6.91	2.8 ± 7.27	(1.3, 4.3)	**<0.001**
Total FMS score	40.4 ± 11.17	47.3 ± 10.43	6.9 ± 12.61	(4.4, 9.4)	**<0.001**

The bold values represent that *p* - values are statistically significant.

As can be seen from the results of the correlation matrix, BMI was not significantly correlated with either fundamental motor skills or physical fitness. The correlation between sit-and-reach and fundamental motor skills was weak, and the rest of the physical fitness were significantly correlated with fundamental motor skills (see Table 5).

**Table 5:** Correlation analysis at T1 and T2.

		T1				T2			
		BMI	Locomotor	Object control	Total FMS	BMI	Locomotor	Object control	Total FMS
T1	BMI	1	−0.19	−0.13	−0.20	0.59***	0.07	0.13	0.13
	Horizontal jump	−0.10	0.42***	0.06	0.34**	0.04	0.20	0.11	0.20
	Tennis ball throwing	0.05	0.27*	0.41***	0.39**	0.17	0.09	0.34**	0.28*
	Sit-and-reach	−0.07	0.13	0.04	0.12	−0.05	−0.01	−0.04	−0.03
	10-meter shuttle run	0.01	−0.54***	−0.34**	−0.56***	0.03	−0.23*	−0.38**	−0.39**
	Balance beam walking	0.14	−0.37**	−0.05	−0.30**	0.18	−0.33**	−0.16	−0.30**
	Continuous jumping with both feet	0.07	−0.35**	−0.16	−0.33**	0.01	−0.14	−0.2	−0.21
T2	BMI	0.59***	0.01	0.01	0.01	1	−0.02	0.04	0.00
	Horizontal jump	−0.08	0.37**	0.19	0.36**	−0.15	0.47***	0.33**	0.49***
	Tennis ball throwing	−0.11	0.38**	0.17	0.36**	−0.11	0.32**	0.30**	0.39***
	Sit-and-reach	−0.12	0.04	−0.13	−0.03	−0.07	−0.15	−0.23*	−0.24*
	10-meter shuttle run	0.14	−0.38**	−0.27*	−0.41***	−0.03	−0.39***	−0.24*	−0.39**
	Balance beam walking	0.20	−0.24*	0.01	−0.18	0.16	−0.28*	−0.13	−0.24*
	Continuous jumping with both feet	0.04	−0.22	−0.22	−0.27*	0.07	−0.35**	−0.28*	−0.39**

* for *p* < 0.05, ** for *p* < 0.01, *** for *p* < 0.001.

### 3.2. Cross-lagged model results

On the basis of the correlation analysis, the cross-lagged model was used to explore the longitudinal relationship between physical fitness and fundamental motor skills. The results showed that age and gender had significant effects on both, while BMI did not. As a result, only age and gender were included as control variables in the model. The model had a good degree of fit: χ^2^ = 120.73, df = 84, χ2/df = 1.44, *p* > 0.05, RMSEA = 0.067, CFI = 0.951, TLI = 0.931, SRMR = 0.049.

**Figure 1:**
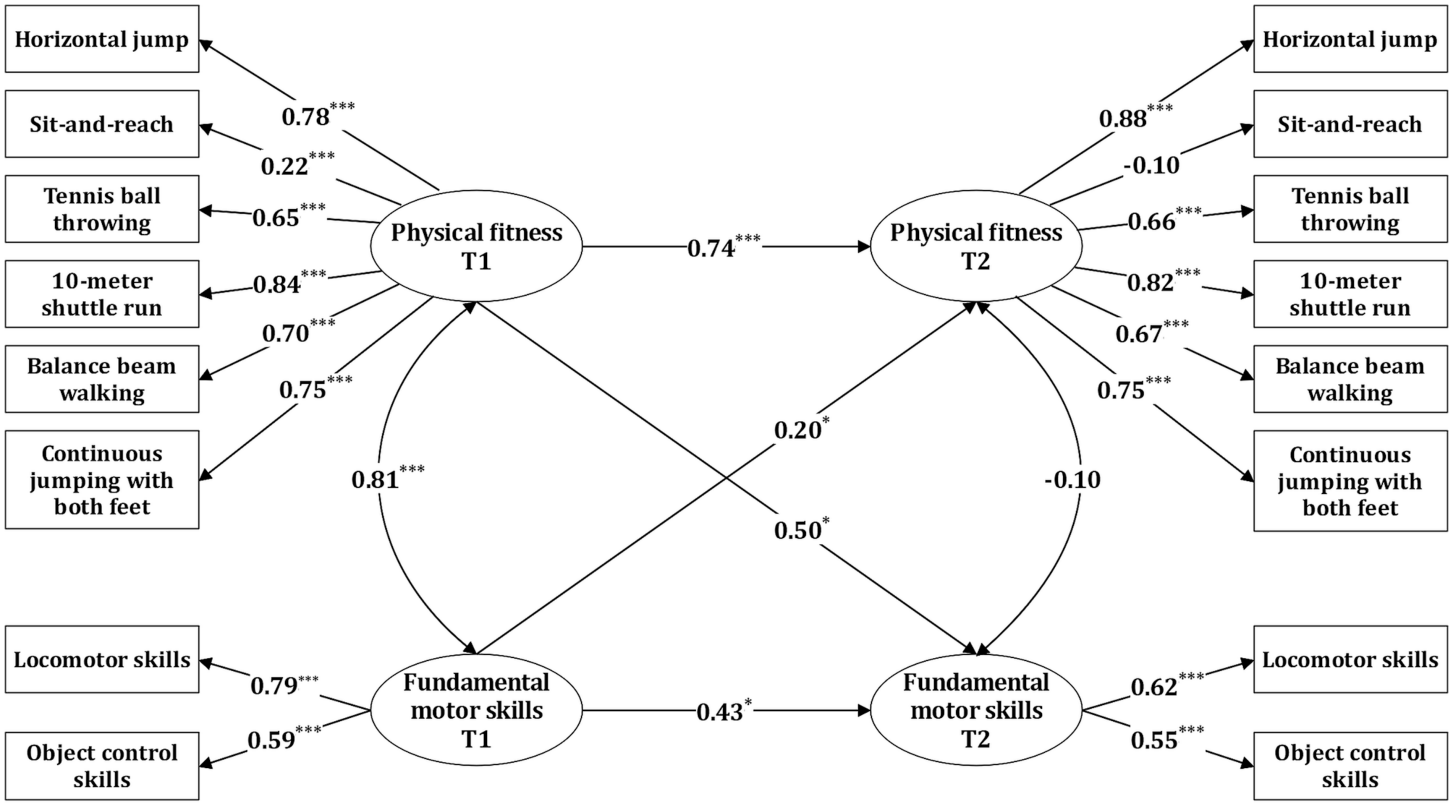
Cross-lagged model results for physical fitness and fundamental motor skills.

Figure 1 showed that T1 physical fitness effectively predicted T2 physical fitness (β = 0.74, SE = 0.086, *p* < 0.001), and similarly, T1 fundamental motor skills effectively predicted T2 fundamental motor skills (β = 0.43, SE = 0.212, *p* = 0.043), In terms of the β coefficient, physical fitness has greater stability compared to fundamental motor skills. After controlling for the effects of age, gender, and autoregressive effects, T1 fundamental motor skills effectively predicted T2 physical fitness (β = 0.20, SE = 0.094, *p* = 0.034). Among the two dimensions of fundamental motor skills, the factor loadings for locomotor skills were higher than those for object control skills. Furthermore, T1 physical fitness also effectively predicted T2 fundamental motor skills (β = 0.50, SE = 0.212, *p* = 0.017). In physical fitness, the factor loadings of sit-and-reach were lower than those of the other dimensions.

## 4. Discussion

This study used a cross-lagged model to investigate the bidirectional relationship between physical fitness and fundamental motor skills in preschoolers. In doing so, it compensated for the shortcomings of previous cross-sectional studies that could determine the magnitude but not the direction of the association, and provided new evidence for the relationship. The findings support our proposed research hypothesis that preschoolers’ fundamental motor skill levels could predict physical fitness (and vice versa) 6 months later.

To control the extraneous variables in the subsequent cross-lagged model, this study first examined the demographic variables of age, gender, and BMI. The results showed that age and gender had a significant effect on physical fitness and fundamental motor skills, while BMI did not. When cross-sectional and longitudinal data were combined, all indicators except BMI increased with age, which is consistent with previous studies (Yang et al., 2015; Ning et al., 2016b; Tu et al., 2021), and with the growth and development patterns of preschoolers. Boys outperformed girls in both object control skills and tennis ball throwing, whereas girls excelled in sit-and-reach. The TGMD-3 also found gender differences in fundamental motor skills when establishing regional norms in the United States (Ulrich, 2019) and Shanghai, China (Diao et al., 2018), thereby setting separate object control skill norms for boys and girls. Previous studies suggest that such gender differences are not biological (Goodway et al., 2021) but are associated with family, environmental, and sociocultural factors (Krombholz, 2006; Spessato et al., 2012; Iivonen and Sääkslahti, 2014). For example: parents tend to encourage boys to participate in ball games, and girls in sports related to gymnastics, dance, or role-play. As a result, boys and girls have different sporting experiences that cause them to perform differently in tennis ball throwing and sit-and-reach. The analysis in this study revealed no association between BMI and either physical fitness or fundamental motor skills. This contradicts previous studies that found a negative relationship between BMI and fundamental motor skills, particularly locomotor skills (Southall et al., 2004; Zhou, 2020). This is because preschoolers need to overcome gravity to coordinate their body movements; overweight and obese children find this more challenging, resulting in lower locomotor skills scores. A Chinese study that investigated the effect of weight on the physical fitness of children aged 5–6 found that the group with normal body weight had a higher level of physical fitness than the under-and overweight groups (Xu et al., 2015). The small number of overweight and obese children in this study’s sample (only 5%) may explain why BMI did not correlate with other indicators.

The cross-lagged results for fundamental motor skills and physical fitness in preschoolers revealed that T1 fundamental motor skills effectively predicted T2 physical fitness. This aligns with several previous longitudinal studies, which found that proficient fundamental motor skills in early childhood had a positive effect on future physical development, including cardiopulmonary endurance, and muscle endurance and strength (Hands, 2008; Fransen et al., 2014; Vlahov et al., 2014; Lima et al., 2019). Fransen et al. (2014) investigated fundamental motor skills, physical fitness and participation in physical activity among 6-year-olds in a two-year follow-up study, and found that children with low levels of movement rarely participated in physical activities. This resulted in a lack of opportunity to develop their fundamental motor skills and physical fitness, which in turn prevented them from catching up with their more active peers. Most of the studies discussed primarily examined the effects of locomotor and stability skills on physical fitness. Our study found further proof of the important role of object control skills in the development of physical fitness. Although the factor loadings for object control skills (0.59) were lower than those for locomotor skills (0.79), this might be related to the composition of the physical fitness test items and the short follow-up period. It does not imply that object control skills are less important than locomotor skills. Vlahov et al. (2014) found in their 11-year follow-up study that object control skills in preschoolers were more predictive of physical fitness in high school than locomotor skills. Despite our study’s shorter follow-up period (6 months), it provides evidence that fundamental motor skills are equally predictive of physical fitness in the short term.

The study also discovered that T1 physical fitness effectively predicted T2 fundamental motor skills, which does not support Stodden’s hypothesis of a unidirectional relationship between physical fitness and fundamental motor skills in early childhood (Stodden, 2008). Two longitudinal studies also found that the performance of 6-year-old children in grip, horizontal jump, and the 1-mile walk could predict fundamental motor skills at age 9 (dos Santos et al., 2018; Henrique et al., 2018). Wang et al. (2019) demonstrated that physical fitness explained 15–16 percent of motor skill variance. All these studies provide evidence of a bidirectional relationship between fundamental motor skills and physical fitness. Among the six physical fitness in this study, the factor loadings for flexibility, measured by sit-and-reach, were substantially lower than those of other fitness. This result aligns with previous studies, Coppens et al. (2019) found that flexibility in 8-year-old children did not predict fundamental motor skill levels two years later. Although flexibility is structurally less correlated with motor skills from a theoretical perspective, its actual impact needs to be confirmed by further studies, as current research is limited. Cattuzzo et al. (2016) highlighted that the interrelationship between fundamental motor skills and physical fitness is closely related, as both require substantial neuromuscular control to achieve efficient and coordinated motor performance. Furthermore, the two are related both directly, through neuromuscular function, and indirectly, through participation in physical activities. Therefore, Fundamental motor skills and physical fitness mutually enhance each other in early childhood, and both should be the focus of sports promotion in preschools.

### 4.1. Research limitations and prospects

This study contained some limitations that should be addressed in future research. Firstly, it utilized a limited number of variables; similar studies usually include a cardiorespiratory test, such as the 20-meter shuttle run, when measuring physical fitness in preschoolers. Additionally, fundamental motor skills include stability skills in addition to locomotor and object control (Barnett et al., 2016). Future studies are recommended to include more variables to explore the role of stability skills and their impact on physical fitness. In addition, sample size is considered important in longitudinal studies. To ensure acceptable attrition rates and account for the rapid growth and development during preschool years, this study used a six-month follow-up interval. However, it was not determined whether this interval was optimal for observing the developmental patterns of both variables. To more comprehensively reveal the developmental trends of the variables and the patterns of the relationships between them, future studies could obtain more accurate relationships between the development of physical fitness and fundamental motor skills in preschoolers by expanding the sample size and extending the duration and number of follow-ups.

### 4.2. Practical applications

Educators should take a balanced view when evaluating the role of fundamental motor skills in pre-school physical education. This study indicates a bidirectional predictive relationship between fundamental motor skills and physical fitness; in fact, physical fitness plays an even stronger predictive role in the development of fundamental motor skills. This two-way relationship contrasts with older perspectives, notably the Stodden’s theoretical model, which posited fundamental motor skills as the sole basis for physical fitness in preschoolers. Relying solely on such a model can result in an imbalanced curriculum that overemphasizes motor skills, hindering well-rounded development in young children.

Moreover, educators should continuously expand their understanding of both fundamental motor skills and physical fitness for preschoolers. Initial surveys from this study revealed that kindergarten teachers in China often have a narrow view, associating fundamental motor skills mainly with actions like “walking, running, jumping, throwing, crawling, climbing, and scaling.” This focus predominantly on locomotor skills overlooks the comprehensive framework of fundamental motor skills, which includes three key dimensions: locomotor, object control, and stability (Barnett et al., 2016). Additionally, in terms of physical fitness, strength, speed, and endurance should not be the focus for preschoolers who lack the necessary physical foundation; instead, attributes related to rapid neurological development such as agility, balance, and coordination should be emphasized (Istvan et al., 2013).

## 5. Conclusion

Physical fitness and fundamental motor skills have been found to exhibit a bidirectional predictive effect in preschoolers. Flexibility, compared to other forms of physical fitness, shows a weaker prediction, while locomotor skills predict more effectively than object control skills. This study provides new evidence for Stodden’s theoretical model, and underscoring the importance of simultaneous learning of physical fitness and fundamental motor skills during preschool years to foster the health and development of young children.

## Data availability statement

The original contributions presented in the study are included in the article/supplementary material, further inquiries can be directed to the corresponding author.

## Ethics statement

The studies involving humans were approved by Ethics Committee of Nantong University. The studies were conducted in accordance with the local legislation and institutional requirements. Written informed consent for participation in this study was provided by the participants’ legal guardians/next of kin.

## Author contributions

JC: Writing – original draft. WS: Writing – review & editing, Methodology. XZ: Writing – review & editing. HL: Writing – review & editing. DL: Writing – review & editing.

## Funding

The author(s) declare that no financial support was received for the research, authorship, and/or publication of this article.

## Conflict of interest

The authors declare that the research was conducted in the absence of any commercial or financial relationships that could be construed as a potential conflict of interest.

## Publisher’s note

All claims expressed in this article are solely those of the authors and do not necessarily represent those of their affiliated organizations, or those of the publisher, the editors and the reviewers. Any product that may be evaluated in this article, or claim that may be made by its manufacturer, is not guaranteed or endorsed by the publisher.
